# Perinatal death in Northern Uganda: incidence and risk factors in a community-based prospective cohort study

**DOI:** 10.1080/16549716.2020.1859823

**Published:** 2021-01-15

**Authors:** Anna Agnes Ojok Arach, James K. Tumwine, Noeline Nakasujja, Grace Ndeezi, Juliet Kiguli, David Mukunya, Beatrice Odongkara, Vincentina Achora, Justin B. Tongun, Milton W. Musaba, Agnes Napyo, Thorkild Tylleskar, Victoria Nankabirwa

**Affiliations:** a Department of Nursing and Midwifery, Faculty of Health Sciences, Lira University, Lira, Uganda; b Department of Paediatrics and Child Health, School of Medicine, Makerere University College of Health Sciences, Kampala, Uganda; c Department of Psychiatry, School of Medicine, Makerere University College of Health Sciences, Kampala, Uganda; d Department of Community Health and Behavioural Sciences, School of Public Health, Makerere University, College of Health Sciences, Kampala, Uganda; e Department of Research, Sanyu Africa Research Institute, Mbale, Uganda; f Department of Public Health, Busitema University Faculty of Health Sciences, Mbale, Uganda; g Department of Paediatrics and Child Health, Gulu University, Gulu, Uganda; h Department of Obstetrics and Gynaecology, Gulu University, Gulu, Uganda; i Department of Paediatrics and Child Health, University of Juba, Juba, South Sudan; j Department of Obstetrics and Gynaecology, Busitema University Faculty of Health Sciences, Mbale, Uganda; k Centre for International Health, University of Bergen, Bergen, Norway; l Department of Epidemiology and Biostatistics, School of Public Health, Makerere University College of Health Sciences, Kampala, Uganda; m Centre for Intervention Science and Maternal Child Health (CISMAC), Centre for International Health, University of Bergen, Bergen, Norway

**Keywords:** Perinatal death, perinatal mortality, early neonatal deaths, risk factors, Uganda

## Abstract

**Background**: Perinatal mortality in Uganda remains high at 38 deaths/1,000 births, an estimate greater than the every newborn action plan (ENAP) target of ≤24/1,000 births by 2030. To improve perinatal survival, there is a need to understand the persisting risk factors for death.

**Objective**: We determined the incidence, risk factors, and causes of perinatal death in Lira district, Northern Uganda.

**Methods**: This was a community-based prospective cohort study among pregnant women in Lira district, Northern Uganda. Female community volunteers identified pregnant women in each household who were recruited at ≥28 weeks of gestation and followed until 50 days postpartum. Information on perinatal survival was gathered from participants within 24 hours after childbirth and at 7 days postpartum. The cause of death was ascertained using verbal autopsies. We used generalized estimating equations of the Poisson family to determine the risk factors for perinatal death.

**Results**: Of the 1,877 women enrolled, the majority were ≤30 years old (79.8%), married or cohabiting (91.3%), and had attained only a primary education (77.7%). There were 81 perinatal deaths among them, giving a perinatal mortality rate of 43/1,000 births [95% confidence interval (95% CI: 35, 53)], of these 37 were stillbirths (20 deaths/1,000 total births) and 44 were early neonatal deaths (23 deaths/1,000 live births). Birth asphyxia, respiratory failure, infections and intra-partum events were the major probable contributors to perinatal death. The risk factors for perinatal death were nulliparity at enrolment (adjusted IRR 2.7, [95% CI: 1.3, 5.6]) and maternal age >30 years (adjusted IRR 2.5, [95% CI: 1.1, 5.8]).

**Conclusion**: The incidence of perinatal death in this region was higher than had previously been reported in Uganda. Risk factors for perinatal mortality were nulliparity and maternal age >30 years. Pregnant women in this region need improved access to care during pregnancy and childbirth.

## Background

Perinatal mortality is a major public health concern worldwide with estimated 2 million stillbirths and 1.8 million early neonatal deaths reported in 2019 [1]. Most of these deaths (98%) occur in low- and middle-income countries (LMIC), with sub-Saharan Africa and South Asia being the most affected [2]. A recent meta-analysis of data from sub-Saharan Africa reported an overall perinatal mortality rate of 34.7 per 1,000 births [3]. This is higher than the global estimate of 26.7 deaths per 1,000 births [1] and doubles the perinatal mortality rate of 13.6 per 1,000 births reported in Georgia, a country with the highest perinatal mortality in Europe [4].

The effects of perinatal death are so devastating for mothers and their families, it is associated with long-lasting economic, psychological and social consequences [5–7]. Several studies have drawn attention to the risk factors for perinatal death [8–14]. However, most of these studies were hospital-based or in an urban/peri-urban setting, excluding home births. Moreover, the incidence of perinatal death in rural areas is higher than in peri/urban areas [10,15,16]. Furthermore, data suggest a contextual variation in the magnitude and factors associated with perinatal death, both within and between countries [3,17].

The Every Newborn Action Plan (ENAP) aims to reduce stillbirths to <10 per 1,000 total births and early neonatal deaths to no more than 10 per 1,000 live births by 2035 [18]. Although this target has been met in 94 high-income countries, the majority of the African countries, including Uganda, need to half the rates to reach the target [19]. While the estimated global perinatal deaths have reduced by about 20% over the last 4 years [1,20], Uganda’s perinatal mortality of 38 deaths per 1,000 births has stagnated over the last 5 years [21,22]. This is above the regional estimates for Eastern Africa (34.5 per 1,000 births) and Western Africa (35.7/1,000 births) [3]. Hence, there is an urgent need for reduction in perinatal mortality in this region. The realization of a perinatal mortality reduction, however, is a complex issue that does not only involve improvement of the accessibility and quality health care [3] but also on improving nutrition, sanitation and education [23,24].

For the reduction of perinatal mortality, a reliable perinatal registration data is essential [10]. However, in rural settings like Northern Uganda, childbirth also regularly takes place outside health facilities and therefore are not reflected in the vital statistics [25,26]. Hence, the incidence, causes and risk factors for perinatal death remain unknown. Therefore, this study aimed to determine the incidence, risk factors, and causes of perinatal death at a community level in order to provide context-specific information for planning interventions to reduce perinatal mortality.

## Methods

### Study design

This was a prospective cohort study among pregnant women recruited at ≥28 weeks and followed up for the first 50 days of life. It was nested in the Survival Pluss trial (NCT0260505369), a cluster-randomized community-based trial. The more detailed description of this study can be found in a previous publication [27]. All pregnant women in the study were identified by community volunteers and followed up until 1 week after birth.

### Study setting

The study was conducted in Lira, a district in Northern Uganda, between January 2018 and March 2019. Lira district has 3 administrative counties, 13 sub-counties, 89 parishes and 751 villages. The district had a population of 410,000 in 2014 [28], served by 31 healthcare facilities, including one referral hospital, 3 healthcare centres with operation rooms, 17 healthcare centres with maternity ward but no surgical facilities, and 10 healthcare centres with only out-patient services (dispensary). The main economic activity in the region is subsistence farming.

The study was carried out in 3 sub-counties, Aromo, Agweng and Ogur. These sub-counties were chosen based on the poor maternal and perinatal indicators and the location in a rural and hard-to-reach area of the district.

### Study participants

Participants were identified by 250 community female volunteers who had received training on ethical conduct and record keeping. They contacted the research team via mobile communication whenever they identified a pregnant woman in their communities. A research assistant, accompanied by the community volunteer, then visited the pregnant woman at home; she was included if she was found to be at least 28 weeks pregnant, resident in the study area and willing to participate in the study. The gestational age was determined based on the last normal menstrual period. Participants were recruited irrespective of antenatal care utilization. Follow-up phone calls and visits were made to ensure that the mothers were visited within 24 hours after childbirth and 7 days postpartum. During recruitment, pregnant women who were likely to leave the study area before the end of 6 months and those with psychiatric illness were excluded.

### Sample size

For this study, the required sample size of 1,812 was estimated using Fleiss statistical methods for rates and proportions [29]. It was assumed that 35% of the pregnant women who experienced a perinatal death had no formal education and 28% with perinatal death had a secondary education [30]. This estimation factored in 0.05 alpha, 80% power and 10% non-response.

### Data collection

A team of 42 trained research assistants, fluent in the local language *Lango* collected the data using a standardized questionnaire in face-to-face interviews conducted at the participant’s home. The standardized questionnaire, with structured questions on demographic, socio-economic and current pregnancy was administered at recruitment. The sections on birth and perinatal survival status were administered within 24 hours after childbirth and at 7 days postpartum.

Research assistants (university graduates) underwent a one-week intensive training on data collection procedures and tools before deployment in the field. This training was facilitated by doctors, nurses and a data analyst. The research assistants that were in two groups lived within the community in Aromo and Agweng sub-counties. They were given mobile cell phones and motorcycles to make timely follow-up visits. Data were checked on a daily basis by the coordinators and supervisors for completeness and consistency before submission.

A verbal autopsy was carried out in cases of perinatal death, using standard verbal autopsy questionnaires developed by the WHO [31]. A verbal autopsy was taken within 2 weeks if relatives felt able and willing to provide information. Deaths were grouped according to timing; if it occurred during the antepartum period, intrapartum period or in the early neonatal period. The cause of death was assigned by two paediatricians after independently reviewing the collected data. A consensus was reached on the verbal autopsy data collected to determine the probable cause of death, which were later assigned by one of the authors to the new International Classification of Death – Perinatal Mortality (ICD-PM) grouping [32]. All data collection tools were translated in *Lango*, pretested, and adjusted as necessary.

### Study variables

The primary study outcome for this study was perinatal death. According to the WHO definition, a stillbirth was a baby born with a gestation of 28 weeks or more and an early neonatal death was a live-born baby that died within the first 7 days of life. Perinatal death was used as an umbrella term for both stillbirth and early neonatal death [33]. The birth weight of stillborn infants was not used to determine gestational age, as this was culturally unacceptable in the study area. Therefore, gestational age was determined based on the last normal menstrual period. The perinatal mortality rate was defined on the WHO definition as the number of perinatal deaths per 1,000 births included in the study (≥28 weeks) [18]. The stillbirth rate was defined as the number of stillbirths per 1,000 total births [18]. The early neonatal death rate was the probability that a child born alive within the study period died during the first 7 days after birth, expressed per 1,000 live births [34]. Twin pregnancies were counted as one (last twin), irrespective of the number who died.

An asset-based wealth index was used to estimate the economic status of the participant’s household, using the principal component analysis. The ‘wealth index’ was computed using the first principal component and based on the availability of nine different household assets. The wealth index was later reduced into three groups; lowest 40%, middle 40% and top 20%. Maternal age was categorized as <20 years, between 20 and 30, and >30 years. Marital status was regrouped as married or unmarried. Maternal education was categorized as no education, only primary education, and secondary education or higher education. Parity (at enrolment) was defined as the number of deliveries a woman had had and grouped as Para 0 (nullipara), Para 1–4 (multipara) and Para 5+ (Grand multipara). Antenatal care utilization was collected as ‘yes/no’ at enrolment. Obesity was defined as a body mass index (BMI) >30 kg/m2. Participants’ place of residence was included to assess for the difference in perinatal mortality between the two administrative divisions (sub-counties). A variable representing the intervention or the control arm of the parent trial was included in the analysis as a potential confounder.

### Statistical analysis

Data were collected using an android-based mobile application (Open Data Kit: https://opendatakit.org) and analysed using STATA version 14.0 (StataCorp; College Station, TX, USA). Categorical variables were summarized as proportions and continuous variables as means with their standard deviations as appropriate.

We used generalized estimating equations of the Poisson family, with a log link, taking into account clustering, and assuming an exchangeable correlation to calculate the risk ratios estimating the magnitude of any associations between exposure variables and perinatal death. Based on scientific literature and biological plausibility, we included the following factors into our model: maternal age, marital status [10,11], maternal education, wealth index, antenatal care attendance [9], parity [11,13,14], intervention of the community trial [12] and maternal obesity [15,35]. We also included the place of residence to assess whether perinatal death varied in the two sub-counties represented in our study. All the variables in the model were assessed for collinearity, which was considered present if the variables had a variance inflation factor (VIF) of >10. In situations of collinearity, we retained the variable with the greater biological plausibility. Multivariable regression analysis was used to take into account potential confounding.

## Results

### Incidence of perinatal death

**Figure 1.**
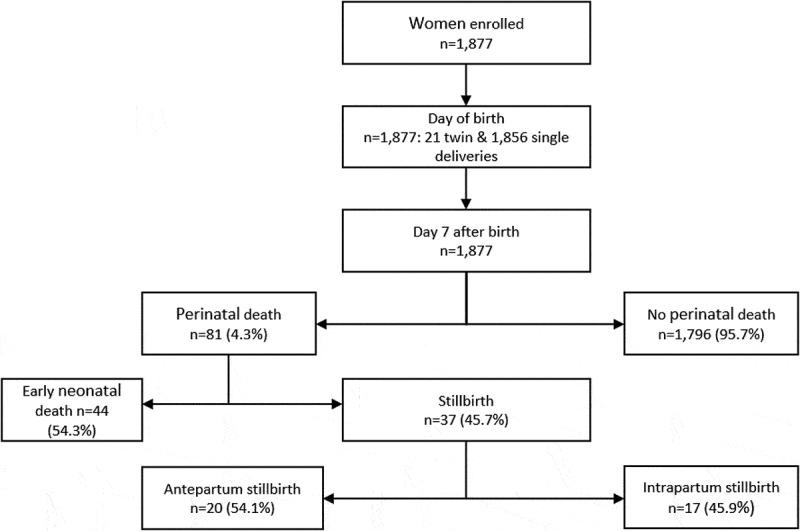
Flow chart of enrolment and follow-up of participants

There were 81 perinatal deaths, resulting in perinatal mortality rate of 43 per 1,000 pregnancies (95% CI: 35, 53), constituted by 37 stillbirths (20 per/1,000 total births) and 44 early neonatal deaths (23 per/1,000 live births). From the 37 stillbirths, there were 20 (54.1%) antepartum and 17 (45.9%) intrapartum deaths (Figure 1). Of the 81 perinatal deaths, two-thirds, 53 (65.4%), were health facility births.

### Risk factors for perinatal death

Table 1 shows the risk factors associated with perinatal death. Nulliparous women had more than twice the risk of experiencing a perinatal death compared to multiparous women (incidence risk ratio IRR: 2.7 [95% CI 1.3, 5.6]). Women over the age of 30 years were more likely to experience a perinatal death than those between 20 and 30 years of age (adjusted IRR: 2.5 [95% CI: 1.1, 5.8]). The risk of perinatal death was higher among women who lived in Agweng sub-county compared to those in Aromo (adjusted IRR: 1.7 [95% CI: 1.1, 2.6]). Other factors, including maternal education, wealth index, obesity, marital status, ANC at enrolment and arm allocation in the parent trial were not associated with perinatal death.

**Table 1.** Characteristics of pregnant women in Lira, Uganda, by perinatal death and crude incidence risk ratio (IRR) and adjusted IRRs of perinatal death

	Total *N* = 1,877 *n* (%)	Perinatal death *N* = 81 *n* (%)	No perinatal death *N* = 1,796 *n* (%)	Crude incidence risk ratio (IRR) [95% CI]	Adjusted incidence risk ratio (IRR) [95% CI]
Maternal education					
None	248 (13.2)	9 (11.1)	239 (13.3)	1	1
Primary	1,459 (77.7)	63 (77.8)	1,396 (77.7)	1.2 [0.5, 2.6]	1.1 [0.5, 2.4]
Secondary or higher	170 (9.1)	9 (11.1)	161 (9.0)	1.4 [0.6, 3.4]	1.0 [0.4, 2.9]
Maternal age					
<20 years	510 (27.2)	32 (39.5)	478 (26.6)	2.1 [1.4, 3.1]	1.1 [0.5, 2.1]
20–30	988 (52.6)	30 (37.0)	958 (53.3)	1	1
>30 years	379 (20.2)	19 (23.5)	360 (20.0)	1.7 [1.0, 2.9]	**2.5 [1.1, 5.8]**
Marital status					
Single	161 (8.7)	7 (8.6)	157 (8.7)	1	1
Married	1,713 (91.3)	74 (91.4)	1,639 (91.2)	1.0 [0.5, 1.9]	1.2 [0.6, 2.2]
Parity at enrolment					
Nullipara	533 (28.4)	37 (45.7)	496 (27.6)	2.3 [1.6, 3.4]	**2.7 [1.3, 5.6]**
Between 1 and 4	954 (50.8)	29 (35.8)	925 (51.5)	1	1
5 and above	390 (20.8)	15 (18.5)	375 (20.9)	1.3 [0.7, 2.2]	0.6 [0.3, 1.3]
Wealth index					
Lowest 40%	837 (44.6)	32 (39.5)	805 (44.8)	1	1
Middle 40%	665 (35.4)	31 (38.3)	634 (35.3)	1.2 [0.9, 1.8]	1.3 [0.9, 2.0]
Top 20%	375 (20.0)	18 (22.2)	357 (19.9)	1.2 [0.8, 1.9]	1.4 [0.8, 2.2]
Sub-county (place of residence)					
Aromo	1,039 (55.4)	35 (43.2)	1,004 (55.9)	1	1
Agweng	838 (44.6)	46 (56.8)	792 (44.1)	1.6 [1.1, 2.5]	**1.7 [1.1, 2.6]**
ANC at enrolment					
No	396 (21.1)	19 (23.5)	377 (21.0)	1.2 [0.8, 1.8]	1.3 [0.9, 2.0]
Yes	1,481 (78.9)	62 (76.5)	1,419 (79.0)	1	1
Maternal obesity					
No	1,590 (84.7)	66 (81.5)	1,524 (84.9)	1	1
Yes	287 (15.3)	15 (18.5)	272 (15.1)	1.3 [0.7, 2.2]	1.2 [0.7, 2.2]
Parent trial allocation					
Control	882 (47.0)	32 (39.5)	850 (47.3)	1	1
Intervention	995 (51.0)	49 (60.5)	946 (52.7)	1.4 [0.9, 2.1]	1.4 [0.9, 2.1]

From the 1,877 participants that were followed up within 24 hours after birth, there were 21 twin births and 1,856 single births (Figure 1). Mean maternal age (±SD) was 25 ± 7 years and the mean duration of education was 4 ± 2 years (range 0 to 18 years). The average number of children a participant had had was 3 (range 0 to 12), details are in Table 1.

### Probable causes of perinatal death

The main probable causes of stillbirths were acute intrapartum events 14 (82.4%) and infections 7 (35.0%). The main probable contributors to early neonatal death were birth asphyxia 16 (36.4%) and respiratory failure 16 (36.4%) (Table 2).

**Table 2.** Probable cause of the 81 perinatal deaths among 1,877 women followed up from at least 28 weeks of gestation to 7 days postpartum in Lira, Northern Uganda, 2018–2019

			Place of birth	
Type of perinatal death	Probable cause of death	Number (%)	Home	Health facility
Antepartum stillbirth	Congenital malformations, deformations and chromosomal abnormalities	4 (20.0)	2	2
	Infection	7 (35.0)	3	4
	Acute antepartum event	2 (10.0)		2
	Disorders related to length of gestation and fetal growth	4 (20.0)	3	1
	Antepartum death of unspecified cause	3 (15.0)	2	1
	**Total antepartum**	20	10	10
Intrapartum stillbirth	Trauma	1 (5.8)	1	
	Acute intrapartum events	14 (82.4)	3	11
	Disorder related to fetal growth	2 (11.8)	1	1
	**Total intrapartum**	17	5	12
Early neonatal death	Congenital malformations, deformations and chromosomal abnormalities	3 (6.8)	1	2
	Disorders related to fetal growth	1 (2.3)	1	
	Birth trauma	1 (2.3)	1	
	Complications of intrapartum events (birth asphyxia)	16 (36.4)	5	11
	Infection	3 (6.8)		3
	Respiratory and cardiovascular disorders (respiratory failure)	16 (36.4)	3	13
	Low birth weight and prematurity	2 (4.5)	1	2
	Neonatal death of unspecified cause	2 (4.5)	1	
	**Total early neonatal**	41	13	31

## Discussion

This study found a perinatal mortality rate of 43 per 1,000 births, constituted by a stillbirth rate of 20 per 1,000 total births and early neonatal mortality rate of 23 per 1,000 live births. The risk of experiencing a perinatal death was greater among nulliparous women and women over the age of 30 years and those living in Agweng. Birth asphyxia, respiratory failure, infections and intra-partum events were the most probable contributors to perinatal death.

Perinatal mortality of 43 deaths per 1,000 births and 95% confidence interval of 35, 53 is slightly higher than the previously reported rates of 38 and 41 deaths per 1,000 births noted in 2016 and 2011, respectively [13,21]. This is above the estimated rates of 29, 39 and 29 deaths per 1,000 births for Kenya (2015), Tanzania (2015) and Rwanda (2014), respectively [36–38]. The higher rate could be due to limited access to quality maternal–child healthcare services. From the evidence, Agweng sub-county registered more mortality than Aromo sub-county yet both are adjacent and in a rural setting. The proximity of some health facilities with maternity services made it easier for pregnant women from Aromo to access better health care services than those in Agweng. In the last national demographic health survey, the region was reported to have poor maternal health indicators such as a larger proportion of pregnant women giving birth outside health facilities compared to the national average, 33 vs. 24% [21,26]. This emphasizes that accessibility to maternal and neonatal health care is a key challenge in the northern region which needs to be addressed to reduce perinatal mortality.

The most probable causes of perinatal death were infection, acute intrapartum events, birth asphyxia and respiratory failure. These can be related to complications of prematurity, labour and delivery, where emergency obstetric and newborn care could have managed the complications [12,39]. The findings are consistent with earlier community-based studies in Uganda, Rwanda, Nepal and Ghana [13,40–43], where neonatal asphyxia, infections and prematurity were the major causes of perinatal death. Further studies are required to establish whether sub-standard care factors are contributing to these deaths so that interventions are well focused towards preventing the deaths.

Nulliparous women (at recruitment) have a higher risk of perinatal death compared to multiparous women. Nulliparous women are prone to labour and delivery complications such as obstruction [12], that increase the risk for neonatal asphyxia and perinatal death [44]. Often, childbirth is initially tried at home, often ending up in complications without timely access to the emergency obstetric care services [45]. Our findings are consistent with results from previous studies conducted in Bangladesh, Uganda and Burkina Faso [11,13,14], where nulliparous women had a higher risk of perinatal mortality relative to multiparous women. Similarly, women over the age 30 years at baseline were twice as likely to experience perinatal death compared to those between 20 and 30 years, as in previous studies conducted in Uganda, Nigeria and Ethiopia [13,15,16,43]. A possible explanation for our findings might be that an older maternal age increases the risk of pregnancy complications, such as placenta previa, gestational diabetes, malpositioning/malpresentation and antepartum haemorrhage, which are proximate factors for perinatal death [46]. Pregnant women who are nulliparous and those over 30 years of age could benefit from risk awareness creation and mobilization for timely seeking of the health care services. Home visitation, counselling and mobilization for care during pregnancy were demonstrated to contribute to reduced perinatal death [47].

Whereas earlier studies have linked socioeconomic status to perinatal death [9–11,23], our findings are similar to other studies that found no significant association between perinatal death and maternal education status [13,24,30,48], household wealth index [13,30] and marital status [13,30]. This could be due to the rural setting of the study, and that access to quality maternity care services is more important than other factors. The poverty level in the region is higher than the national average (32.5 versus 21.4%), and therefore the participants overall are poor, resulting in no difference. Although earlier studies suggest that obesity is a predictor of perinatal mortality [15,35], our results showed no such association, but this may be due to the low prevalence of obesity in the study region [49].

### Strengths and limitations

One strength of this study is that it is prospective and community-based, including ‘all’ women in the clusters. Moreover, the study recorded a complete follow-up. This means that the results are likely to reflect a fairly accurate estimate of perinatal mortality. There are also some limitations: gestation age was estimated basing on the last normal menstrual period (LNMP), which has its uncertainties. Post-mortem diagnostics are absent in the study area; therefore, the probable causes of death were based on ‘verbal autopsy’, which is subject to the recall of events before, during and after childbirth which may be incomplete and unreliable. The parent trial could have increased the health facility deliveries and therefore reduced the number of perinatal deaths (the Hawthorne effect) [50].

## Conclusion

The incidence of perinatal death in the northern region was higher than previously reported in Uganda. The risk of perinatal death was greater among nulliparous women and women over the age of 30 years. Birth asphyxia, respiratory failure, infections and intra-partum events were the major probable contributors to perinatal death. The findings of this study suggest that pregnant women in the region need improved access to care during pregnancy and childbirth. Further studies are required to understand whether the sub-standard care factors are contributing to perinatal deaths so that interventions are well targeted.

## Data Availability

The datasets used and/or analysed during the current study are available from the corresponding author on reasonable request.

## Paper context

To achieve the ‘every newborn action plan’ (ENAP) target of reducing stillbirth and early neonatal death, there is a need to provide specific data locally to guide interventions. The findings show that perinatal mortality in Northern Uganda is higher than previously reported. The risk of experiencing a perinatal death was greater among nulliparous women and women >30 years of age. Pregnant women in this region need improved access to care during pregnancy and childbirth.
